# Functional interplay between (p)ppGpp and RNAP in *Acinetobacter baumannii*

**DOI:** 10.1371/journal.ppat.1013795

**Published:** 2025-12-18

**Authors:** Anthony Perrier, Aurélie Budin-Verneuil, Régis Hallez

**Affiliations:** 1 Bacterial Cell cycle & Development (BCcD), Biology of Microorganisms Research Unit (URBM), Namur Research Institute for Life Science (NARILIS), University of Namur, Namur, Belgium; 2 Université de Caen Normandie, Université de Rouen Normandie, Normandie Université, CBSA UR4312, Caen, France; Children's Hospital Boston, UNITED STATES OF AMERICA

## Abstract

The (p)ppGpp-dependent stress response is required for pathogenic bacteria to survive both outside and inside the host but the mechanisms behind this survival are mostly unknown. In this study, we characterize the (p)ppGpp metabolism in the opportunistic pathogen multi-drug-resistant *Acinetobacter baumannii*. We show that two stressful conditions potentially encountered during infection – iron starvation and polymyxin exposure – induce (p)ppGpp production. The absence of (p)ppGpp led to multiple consequences on the physiology of *A. baumannii*, including an increase of surface motility, a decrease in catalase activity, a poor survival upon nutrient starvation, a rapid killing during desiccation and a strong attenuation in a *Galleria mellonella* model of infection. Using a motility suppressor screen, we isolated multiple independent stringent mutations in *rpoB* and *rpoC* that suppress the (p)ppGpp-dependent phenotypes. By combining the suppressor screen with deep sequencing, we isolated dozens of additional mutants, expanding the list of putative stringent RNAP mutations described so far. Furthermore, our transcriptomic data reveal that (p)ppGpp deeply impacts the transcriptional landscape of *A. baumannii* on solid surface to induce many stress-related genes, including catalase and hydrophilins critical for tolerance to desiccation. This work highlights the functional interplay between (p)ppGpp and RNAP in the successful survival of *A. baumannii* in the environment but also during infection.

## Author summary

The bacterial multi-drug resistant (MDR) opportunistic pathogen *Acinetobacter baumannii* has been of rising concern in the last decades, with carbapenem-resistant strains classified as pathogens of critical importance by the World Health Organization. In addition to the threat caused by its MDR status, *A. baumannii* is also known to be highly resistant and tolerant to stressful conditions and can persist for weeks on biotic and abiotic surfaces. In this study we show that the highly conserved stress response driven by the second messenger (p)ppGpp together with RNAP largely contribute to the virulence and the high resilience of *A. baumannii* in adverse conditions. Our work strongly suggests that the contact on solid surface induces (p)ppGpp accumulation, which leads to a transcriptional reprogramming that sets *A. baumannii* in a survival and virulent state.

## Introduction

The stringent response (SR) is one of the mechanisms that facilitate bacterial adaptation to harmful conditions. In 1969, Cashel and Gallant identified two compounds related to the SR, the guanosine tetra-phosphate (ppGpp) and penta-phosphate (pppGpp) collectively referred to as (p)ppGpp [1]. The SR was first referred to the growth arrest of *Escherichia coli* cells experiencing amino acid starvation due to increasing intracellular levels of (p)ppGpp. Now, the SR refers to any response caused by elevated levels of (p)ppGpp by any means [2]. The intracellular levels of (p)ppGpp are mainly regulated by enzymes of the RSH family (RelA/SpoT Homologue). Most bacteria possess one long bifunctional RSH, whereas the β- and γ-Proteobacteria harbor two long RSH proteins, one bifunctional (SpoT) and one monofunctional (RelA). Interestingly, based on *in silico* analyses and experimental evidence, the Moraxellaceae family to which belongs the genus *Acinetobacter* seems to have two long monofunctional RSH, RelA and SpoT displaying respectively functional synthetase and hydrolase activities [3,4]. In addition to long RSH, some bacteria also possess short monodomain RSH with either a synthetase or hydrolase activity, respectively called Small Alarmone Synthetase (SAS) or Hydrolase (SAH).

Since its discovery more than 50 years ago, it has been often hypothesized that (p)ppGpp directly interacts with the bacterial RNA polymerase (RNAP). But it is only during the last decade that substantial progress has been made and two (p)ppGpp binding site (respectively referred to as site 1 and site 2) were mapped on the *E. coli* RNAP. The site 1 is located at the interface between the ω and β’ subunits [5–7] while the site 2 is created by the interaction of the transcription factor DksA with the β’ subunit [8]. The position of the two binding sites strongly suggests an allosteric regulation of the RNAP by (p)ppGpp. Additionally, (p)ppGpp can also bind to other proteins mostly acting as a competitive inhibitor of guanosine nucleotides [9].

A *E. coli* K12 strain devoid of (p)ppGpp synthetase (Δ*relA* Δ*spoT*) – and thus unable to produce (p)ppGpp hereafter referred as (p)ppGpp^0^ – harbors several physiological defects including amino acids auxotrophy. Growing a (p)ppGpp^0^ strain on minimal medium without amino acids allows the selection of spontaneous suppressors, always mapping to the RNAP, frequently in the β or β’ subunit and rarely in σ^70^ (*rpoD*) [10]. Such mutants known as “stringent RNAP” [11] can suppress other (p)ppGpp-related phenotypes.

*Acinetobacter baumannii* is a gram-negative γ-Proteobacterium belonging to the Moraxellaceae family. In 2017, the World Health Organization (WHO) established a list of bacterial pathogens of public health importance and ranked carbapenem-resistant *A. baumannii* (CRAB) amongst the most critical pathogens. More recently in 2024, the list has been revised but CRAB maintained their critical status because of the high mortality following antibiotic-resistant infections as well as the lack of new drug effective against metallo-β-lactamase producing CRAB [12]. Known as a multidrug-resistant opportunistic pathogen, *A. baumannii* is also highly resistant and tolerant to stressful environments [13]. For instance, clinical isolates of *A. baumannii* can persist for months on dry surfaces in an extreme dehydrated state [14]. Intrinsically disordered proteins called hydrophilins were shown to promote tolerance to desiccation [15].

With two long monofunctional RSH enzymes and DksA recently proposed to functionally replace the major stress response sigma factor RpoS [16], *A. baumannii* stands out as unusual and intriguing amongst γ-Proteobacteria. However, studies on (p)ppGpp in *A. baumannii* are still scarce [17,18]. As seen in many other pathogens, (p)ppGpp^0^ strains display virulence defects both in mouse and *Galleria mellonella* infection models [18,19]. Beyond using serine hydroxamate (SHX) as a proxy for amino acid starvation, the specific stresses that trigger the SR in *A. baumannii* are still unknown. Production of (p)ppGpp has also been described to regulate surface motility with distinct outcomes observed between the reference strains ABUW5075 and ATCC17978 [18,19]. This prompted us to further investigate the physiological role of this second messenger in *A. baumannii*.

Here, we describe a third enzyme likely involved in (p)ppGpp metabolism, *ABUW_1957* encoding a SAH capable of hydrolyzing (p)ppGpp *in vivo* both in *E. coli* and *A. baumannii*, although its exact function remains to be elucidated. A thorough phenotyping of RSH mutants highlighted a critical role of (p)ppGpp in the survival of *A. baumannii* cells exposed to nutrient deprivation and desiccation. We also show that iron starvation and exposure to polymyxin B, two stresses possibly encountered during infection, induced (p)ppGpp production. We isolated stringent RNAP mutants that suppress most of the (p)ppGpp-dependent phenotypes, including virulence defects of the (p)ppGpp^0^ strain in the *G. mellonella* model of infection. By combining our suppressor screen with deep sequencing in a Mut-Seq approach [20], we largely expanded our list of candidate missense mutations within *rpoB* and *rpoC* that likely generate stringent RNAP. Finally, we show that ~8% of the entire transcriptome was significantly modified in (p)ppGpp^0^ cells in contact with solid surface and that the expression of the vast majority of these differentially expressed genes was restored in a stringent RNAP suppressor. Together our data demonstrate that RNAP and (p)ppGpp jointly regulate several phenotypical traits critical for the survival in stressful conditions and the virulence of *A. baumannii*.

## Results

### *A. baumannii* harbors three RelA/SpoT homolog enzymes

*A. baumannii* carries two genes coding for long RSH enzymes, *spoT* (*ABUW_0309*; *ABUW_RS01520*) and *relA* (*ABUW_3302*; *ABUW_RS16040*). RelA^Ab^ is a monofunctional synthetase enzyme with a degenerate and inactive hydrolase domain (S+ /H^-^) [18]. However, in contrast to *E. coli* and many other β- and γ-proteobacteria where SpoT is a bifunctional RSH both synthetizing and hydrolyzing (p)ppGpp (S+ /H+ ), SpoT^Ab^ is a monofunctional hydrolase enzyme with a pseudo-synthetase domain regulating its hydrolase activity (S^-^/H+ ) [4].

**Fig 1:**
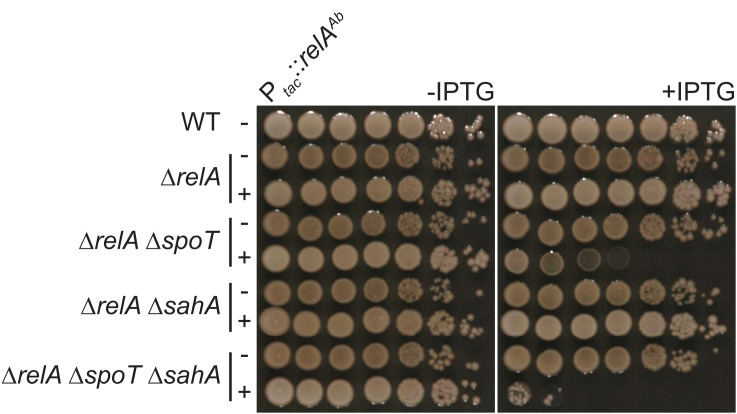
SahA can hydrolyze (p)ppGpp *in vivo* in *A. baumannii.* Viability of *A. baumannii* AB5075 WT, Δ*relA*, Δ*relA* Δ*spoT*, Δ*relA* Δ*sahA,* Δ*relA* Δ*spoT* Δ*sahA* cells carrying a copy of *relA*^*Ab*^ expressed from an IPTG-inducible promoter (P_*tac*_::*relA*^*Ab*^). Overnight cultures were serial diluted (1:10), 5 μL of cells were spotted on LB agar plates with or without IPTG (500 μM) and incubated overnight at 37 °C.

In addition to RelA^Ab^ and SpoT^Ab^, we identified two putative short RSH, one SAH (*ABUW_1957*; *ABUW_RS09520*) and one SAS (*ABUW_0769*; *ABUW_RS03770*). First, we investigated the role of the putative SAH hereafter referred to as SahA. In *E. coli*, *spoT* is essential in an otherwise wild-type (WT) background since (p)ppGpp produced by RelA accumulates to a toxic level in a Δ*spoT* background when grown on complex media. In contrast, *spoT* can be inactivated in a Δ*relA* background. To test whether SahA could hydrolyze (p)ppGpp, a P1 lysate made on *E. coli* MG1655 Δ*relA* Δ*spoT::kan*^*R*^ was transduced in a WT strain of *E. coli* MG1655 harboring a pBAD33 plasmid, either empty or expressing *spoT*^*Ec*^, *spoT*^*Ab*^ or *sahA* under the control of the arabinose-inducible pBAD promoter. As shown in S1A Fig, the endogenous *spoT* gene could be inactivated in the presence of arabinose and one of the three expression vectors (pBAD33-*spoT*^*Ec*^, pBAD33-*spoT*^*Ab*^ or pBAD33-*sahA*) but not with the empty pBAD33 plasmid, strongly suggesting that SahA is indeed able to hydrolyze (p)ppGpp *in vivo*. Then, we tested whether *sahA* and *spoT* could compensate each other as (p)ppGpp hydrolases in *A. baumannii*. For that, we first generated Δ*relA*, Δ*relA* Δ*spoT*, Δ*relA* Δ*sahA* and Δ*relA* Δ*spoT* Δ*sahA* knock-out mutants in *A. baumannii* AB5075*.* Then, a copy of *relA*^*Ab*^ under the control of an IPTG-inducible promoter was introduced in all these mutant strains. Interestingly, induction of *relA*^*Ab*^ in the Δ*relA* Δ*spoT* mutant strain led to a severe growth defect whereas the strain lacking both (p)ppGpp hydrolases (Δ*relA* Δ*spoT* Δ*sahA*) could barely grow when *relA*^*Ab*^ was induced. In contrast, expressing back *relA*^*Ab*^ in strains harboring a functional copy of *spoT –* the single Δ*relA* or the double Δ*relA* Δ*sahA* mutant strains – did not impact the growth (Fig 1). These results further suggest that SahA is, to some extent, able to hydrolyze (p)ppGpp *in vivo*, both in *E. coli* and *A. baumannii*. Nevertheless, despite several attempts with different methods, we failed to generate a single Δ*spoT* mutant in *A. baumannii*, strongly suggesting that SpoT is the main (p)ppGpp hydrolase in *A. baumannii* and that SahA cannot compensate for the loss of *spoT*, at least in the conditions we tested.

Finally, we investigated the role of the putative SAS. The fact that a Δ*relA* Δ*spoT* Δ*sahA* triple mutant in *A. baumannii* was viable already suggested that (p)ppGpp was unlikely produced by this putative SAS, at least in our lab conditions. A whole genome sequencing of this triple mutant excluded the presence of any suppressor mutations. To further investigate the potential (p)ppGpp production *in vivo* by this enzyme, *E. coli* MG1655 WT(hydrolase +) and MG1655 Δ*relA* Δ*spoT* (hydrolase -) strains were transformed with a pBAD33 plasmid, either empty or harboring *relA*^*Ec*^ or *ABUW_0769*, and grown on complex LB medium supplemented with glucose or arabinose. Expressing *relA*^*Ec*^ was toxic in the Δ*relA* Δ*spoT* background, likely because of (p)ppGpp accumulation whereas the expression of *ABUW_0769* did not have any impact on the growth of both strains (S1B Fig). Together with the fact that (p)ppGpp was undetectable in the *A. baumannii* Δ*relA* and Δ*relA* Δ*spoT* mutant strains (S2 Fig), these results suggest that *ABUW_0769* is unlikely to function as a (p)ppGpp synthetase in *A. baumannii*, at least in all the conditions we tested.

### The absence of (p)ppGpp induces pleiotropic effects in *A. baumannii*

It has been previously described that a Δ*relA* mutant in the AB5075 background shows a hyper-motile phenotype and forms elongated cells in stationary phase [18]. Considering the high phenotypic heterogeneity among *A. baumannii* strains and also within the same strain in different labs [21] we investigated several known (p)ppGpp-dependent phenotypes in our mutants.

**Fig 2:**
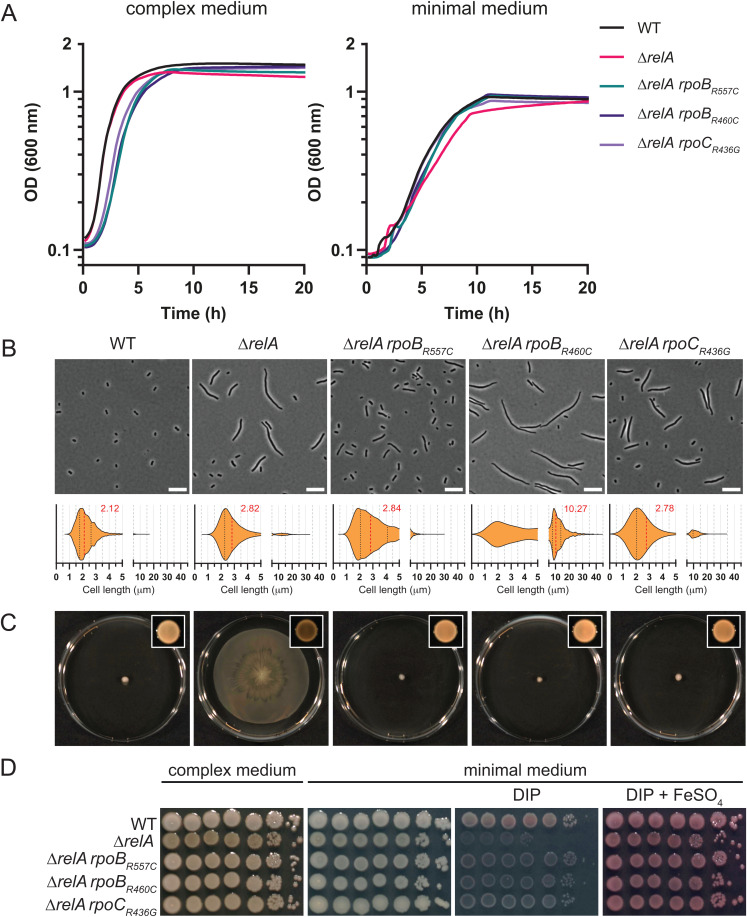
The pleiotropic defects of Δ*relA* are suppressed by mutations in the RNAP. (**A**) Representative growth of WT, Δ*relA*, Δ*relA rpoB*_*R557C*_, Δ*relA rpoB*_*R460C*_ and Δ*relA rpoC*_*R436G*_ at 37 °C in complex (LB) or minimal synthetic (M9X) medium for 20 h in 96-well plates. The growth curves represent the average of 3 technical replicates. (**B**) Microscopic analysis of WT, Δ*relA*, Δ*relA rpoB*_*R557C*_, Δ*relA rpoB*_*R460C*_ and Δ*relA rpoC*_*R436G*_ cells grown overnight at 37 °C in liquid complex medium. A representative picture (scale bar = 10 μm) is shown for each strain accompanied by a violin plot showing cell size distribution; the median size (μm) is indicated in red (between 2348 and 3308 cells were used for each strain). (**C**) Surface motility of cells described in (B) on low agar (0.5%) complex medium. The appearance and the color of the colonies can also be observed on the picture in the box in top right corners. Pictures for both experiments were taken after 24 h incubation at 37 °C. (**D**) Viability of WT, Δ*relA*, Δ*relA rpoB*_*R557C*_, Δ*relA rpoB*_*R460C*_ and Δ*relA rpoC*_*R436G*_ strains on complex (LB) or minimal synthetic (M9X) medium with or without supplementation [DIP (200 μM); FeSO_4_ (50 μM]. Overnight cultures were serial diluted (1:10), 5 μL of cells were spotted on plates and incubated overnight at 37 °C.

First, the growth of the mutants was measured in complex medium (LB) and minimal synthetic medium with xylose as sole carbon source (M9X) and compared to the WT. Surprisingly, we did not find significant differences, except a lower plateau for Δ*relA* and Δ*relA* Δ*spoT* cells grown in LB and a slight growth delay in M9X (Figs 2A and S3A).

Then, we analyzed the cell morphology of bacteria grown overnight in LB. The Δ*relA* and Δ*relA* Δ*spoT* mutants showed heterogenous filamentation with a median of 2.82 and 2.51 μm, respectively, against 2.12 μm for the WT strain (Figs 2B and S3B). In contrast, the Δ*sahA* mutant showed a cell size distribution close to the WT strain with a median of 2.24 μm (S3B Fig). We also looked at surface motility on low agar plates. After 24 h at 37 °C, the Δ*relA* and Δ*relA* Δ*spoT* strains colonized most of the 90 mm Petri dishes, whereas the WT and Δ*sahA* strains did not move at all, with only growth observed around the inoculation site (Figs 2C and S3C). Interestingly, the motility behavior correlates well with the colony aspect of the different strains. The WT and Δ*sahA* strains formed white shiny colonies while the Δ*relA* and Δ*relA* Δ*spoT* strains formed browner colonies on LB agar plates (S3C Fig).

**Fig 3:**
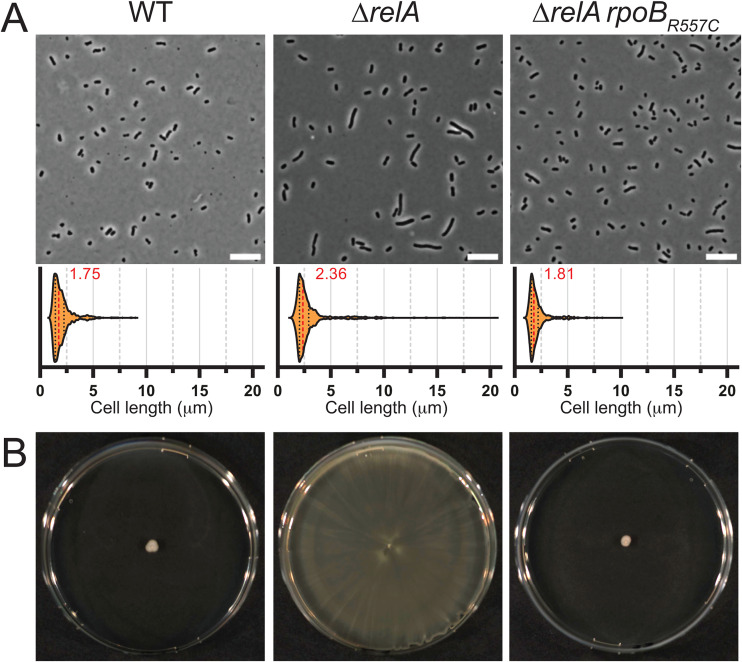
*rpoB_R557C_* is also a suppressor of Δ*rel*A in *A. baumannii* ATCC17978 strain. (**A**) Microscopic analysis of WT, Δ*relA*, Δ*relA rpoB_R557C_* cells grown overnight at 37 °C in liquid LB medium. A representative picture (scale bar = 10 μm) is shown for each strain accompanied by a violin plot showing cell size distribution; the median size (μm) is indicated in red (between 1164 and 1534 cells were used for each strain). (**B**) Surface motility of cells described in (A) on low agar (0.5%) complex medium grown at 37 °C for 24 h.

Since *A. baumannii* is characterized by a wide genetic and phenotypic diversity, a Δ*relA* mutant was also constructed in the well-studied ATCC17978 strain. Similarly to AB5075 Δ*relA*, ATCC17978 Δ*relA* formed an heterogenous population of short and longer cells with a median of 2.36 μm when grown overnight in LB compared to 1.75 μm for the ATCC17978 WT strain (Fig 3A). Likewise, we found that in our conditions, WT cells from the ATCC17978 strain were not motile in contrast to the Δ*relA* mutant which colonized the entire plate in 24 h (Fig 3B).

Finally, we used serial dilution drop assays to investigate the sensitivity of our strains to several stresses described in *E. coli* as linked to (p)ppGpp. S3 Fig shows that the Δ*sahA* strain behaved like the WT strain under all conditions tested. Surprisingly, despite the fact that exposure to SHX – known to induce amino acid starvation – strongly induced (p)ppGpp accumulation (S2 Fig), the strains unable to produce (p)ppGpp (Δ*relA* and Δ*relA* Δ*spoT*) could grow well on synthetic minimal medium M9 with xylose as sole carbon source (M9X), showing that none of these mutants suffered from amino acid auxotrophy. On the contrary, Δ*relA* mutants had severe growth defects on plates containing cerulenin or triclosan (fatty acid starvation), or 2,2′-dipyridyl (DIP) (iron starvation), while the addition of 50 μM of iron alleviated the defects induced by DIP (Figs 2D and S3C).

**Fig 4:**
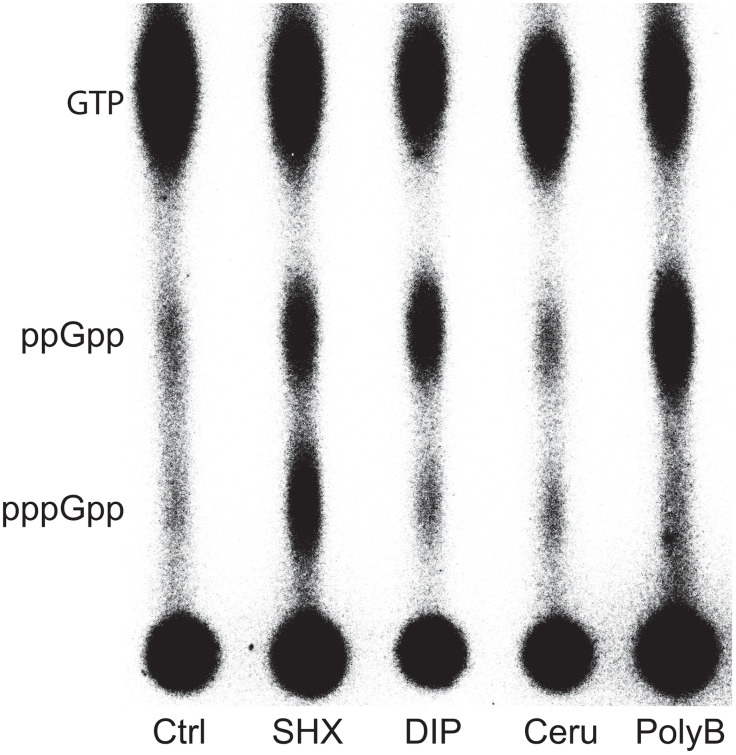
Serine hydroxamate, 2,2’-dipyridyl and polymyxin B induce (p)ppGpp production in *A. baumannii.* Nucleotides extracted from WT cells either non-treated (Ctrl) or exposed to 3.4 mM serine hydroxamate (SHX), 400 μM 2,2’-dipyridyl (DIP), 250 μg/mL cerulenin (Ceru) or 2 μg/mL polymyxin B (PolyB) for 10 min, were analyzed on thin layer chromatography.

In agreement with our drop assay, we found that DIP led to noticeable ppGpp accumulation. However, to our surprise, cerulenin did not induce (p)ppGpp production in our conditions, suggesting that in contrast to what is described in *E. coli*, fatty acid starvation is not a signal triggering (p)ppGpp accumulation in *A. baumannii* (Fig 4). We also tested polymyxin B and, as previously shown in *E. coli* [22], we observed ppGpp accumulation in cells exposed to this last-resort antibiotic used against *A. baumannii* infection (Fig 4).

### Mutations in *rpoB* or *rpoC* suppress the (p)ppGpp-dependent phenotypes

While characterizing the hyper-motility behaviour of Δ*relA* on low agar plates, we inadvertently selected suppressors, which appeared after more than 96 h of incubation at 37 °C as white shiny dots on top of the bacterial lawn of motile cells (S4 Fig). Mutations in *rpoB* or *rpoC* – respectively encoding the β and β‘ subunits of the RNAP – were found by whole genome sequencing in all tested non-motile suppressor candidates (Table 1). In *E. coli*, (p)ppGpp binds to the RNAP to reprogram transcription and mutations in *rpoB* or *rpoC* can induce conformational changes that mimic the effect of (p)ppGpp binding [23]. Moreover, some of these so-called stringent mutations were associated with resistance to rifampicin (Rif^R^), but none of the suppressor mutations that we identified here (*rpoB*_*R460C*_, *rpoB*_*R557C*_ and *rpoC*_*R436G*_) led to a Rif^R^ phenotype.

**Table 1:** List of the putative mutations leading to stringent RNAP in *A. baumannii.*

	WT AA	Position *A. baumannii*	Position *E. coli*	Mutant AA	Hotspot
**RpoB (β)**	**P**	**155**	**153**	**L**	**I**
	**G**	**156**	**154**	**V**	**I**
	**P**	**180**	**178**	**L**	**I**
	V	196	194	G	I
	**G**	**402**	**401**	**V/R**	
	R	403	402	P	
	**R**	**460**	**451**	**H/C/S**	**II**
	**R**	**463**	**454**	**H/C/S**	**II**
	**S**	**521**	**512**	**F**	**II**
	**A**	**541**	**532**	**E**	**II**
	**L**	**542**	**533**	**P**	**II**
	**G**	**543**	**534**	**D/C**	**II**
	**G**	**545**	**536**	**V**	**II**
	**G**	**546**	**537**	**D/C**	**II**
	**A**	**552**	**543**	**R**	**II**
	R	557	548	H/C/S	II
	**R**	**566**	**557**	**P/G**	**II**
	**T**	**572**	**563**	**I**	**II**
	G	575	566	C	II
	**G**	**579**	**570**	**S**	**II**
	D	823	814	E	
	N	1093	1072	K	
	**H**	**1263**	**1244**	**L**	**III**
	T	1274	1255	R	III
	**G**	**1280**	**1261**	**C**	**III**
	**G**	**1286**	**1267**	**C**	**III**
	**Q**	**1287**	**1268**	**K**	**III**
	L	1306	1287	F	III
	**P**	**1336**	**1317**	**L**	**III**
	S	1341	1322	L	III
	**S**	**1351**	**1332**	**P**	**III**
**RpoC (β‘)**	S	331	326	F/Y	IV
	R	342	337	C	IV
	R	436	431	G	IV
	C	459	454	S	IV
	F	466	461	C	IV
	A	499	494	E	IV
	**R**	**785**	**780**	**C**	**V**
	A	789	784	D	V
	A	796	791	E	V
	**R**	**803**	**798**	**L**	**V**
	**R**	**804**	**799**	**L/H**	**V**
	A	1143	1147	D	VI
	**R**	**1144**	**1148**	**C**	**VI**
	V	1236	1240	G	VI
	G	1304	1308	D	VI
	**R**	**1326**	**1330**	**C**	**VI**
	**G**	**1350**	**1354**	**D**	**VI**

Each row represents missense mutations in RpoB or RpoC. Orange indicates mutations that confer Rif^R^ phenotype; blue indicates mutations that have been found by Mut-Seq and/or by Sanger sequencing; bold highlights mutations already found in *E. coli* as RNAP stringent mutants.

**Fig 5:**
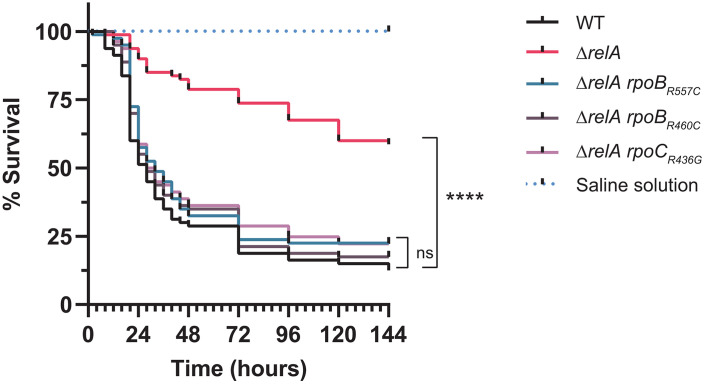
Mutations in *rpoB* and *rpoC* restore the virulence of a Δ*relA* mutant. Kaplan-Meier survival analysis of *Galleria mellonella* larvae infected with the WT, Δ*relA,* Δ*relA rpoB*_*R557C*_, Δ*relA rpoB*_*R460C*_ and Δ*relA rpoC*_*R436G*_ strains. Each curve represents the average of three independent experiment for a total of 80 larvae per condition. All survival curves are not significantly different from the WT survival curve except the one for the Δ*relA* mutant (Log-rank (Mantel-Cox) test; *p* < 0.0001).

Interestingly, these three mutations could suppress most of the (p)ppGpp-dependent phenotypes observed in Δ*relA*, including the motility and the growth defects (Fig 2). In contrast, the cell filamentation observed in stationary phase was still present in all the three suppressors and was even more pronounced in the Δ*relA rpoB*_*R460C*_ strain (Fig 2B). When testing for cerulenin or triclosan sensitivity, only *rpoC*_*R436G*_ failed to suppress the growth defects of Δ*relA* (S3D Fig). More importantly and despite these differences, we found that the three suppressor mutations could fully restore the virulence of *A. baumannii* in the *Galleria mellonella* model of infection. Indeed, the Kaplan-Meier analysis showed that the survival of the larvae infected with each of the suppressors was not significantly different from the WT, while in agreement with previous report [4,18], the larvae infected with Δ*relA* showed a significantly better survival (*p* < 0.0001) (Fig 5).

We also selected motility suppressors for the ATCC17978 Δ*relA* mutant by incubating low agar plates for an extended time, until white shiny colonies appeared on top of the bacterial lawn as observed for the AB5075 strains. By using specific primers that anneal to the *rpoB*_*R557C*_ allele, we were able to fish out 8 positive clones that were then validated by Sanger sequencing. Again, as for AB5075, *rpoB*_*R557C*_ was able to suppress most of the phenotypes displayed by Δ*relA* in the ATCC17978 strain (Fig 3).

**Fig 6:**
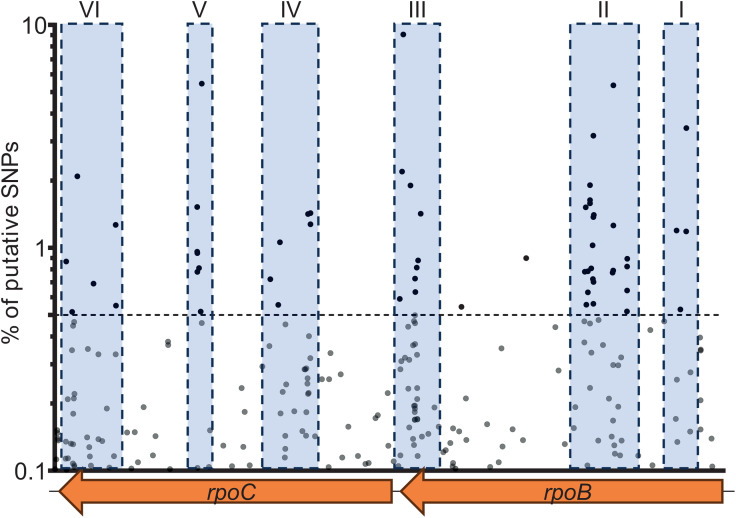
Putative stringent mutations leading to loss-of-motility in Δ*relA* are spread in 6 sub-regions within *rpoB* and *rpoC.* For a given nucleotide position, only SNPs found by Mut-Seq with a percentage ≥ 0.1 are represented. Confidence threshold of 0.5% is represented by the horizontal dashed line. Blue boxes highlight the sub-regions where most SNPs were found.

To check to what extent *rpoB* and *rpoC* are hotspots for the selection of Δ*relA* suppressors, we used an unbiased Mut-Seq approach [20] to map as many mutations as possible in *rpoB* or *rpoC* that suppress the hyper-motility phenotype displayed by Δ*relA*. For that, 284 non-motile Δ*relA* suppressors were selected on low agar complex medium plates as described above. All the clones were tested for rifampicin resistance and eight of them were Rif^R^ (Table 1). Then, the pooled gDNA of these 284 clones was used as a template to amplify *rpoB* and *rpoC* loci and both PCR products were deep sequenced. By using a confidence threshold of 0.5% (see S1 Methods), 38 and 19 putative substitutions – leading to 31 and 17 missense mutations respectively in *rpoB* and *rpoC* – were identified (Fig 6 and Table 1).

Together these data strongly support that the (p)ppGpp-dependent transcriptional reprogramming is responsible for most of the phenotypes observed in (p)ppGpp^0^ cells and suggest that RNAP is a primary (p)ppGpp target in *A. baumannii*.

### A single substitution in *rpoB* can revert the (p)ppGpp-dependent transcriptomic changes

**Fig 7:**
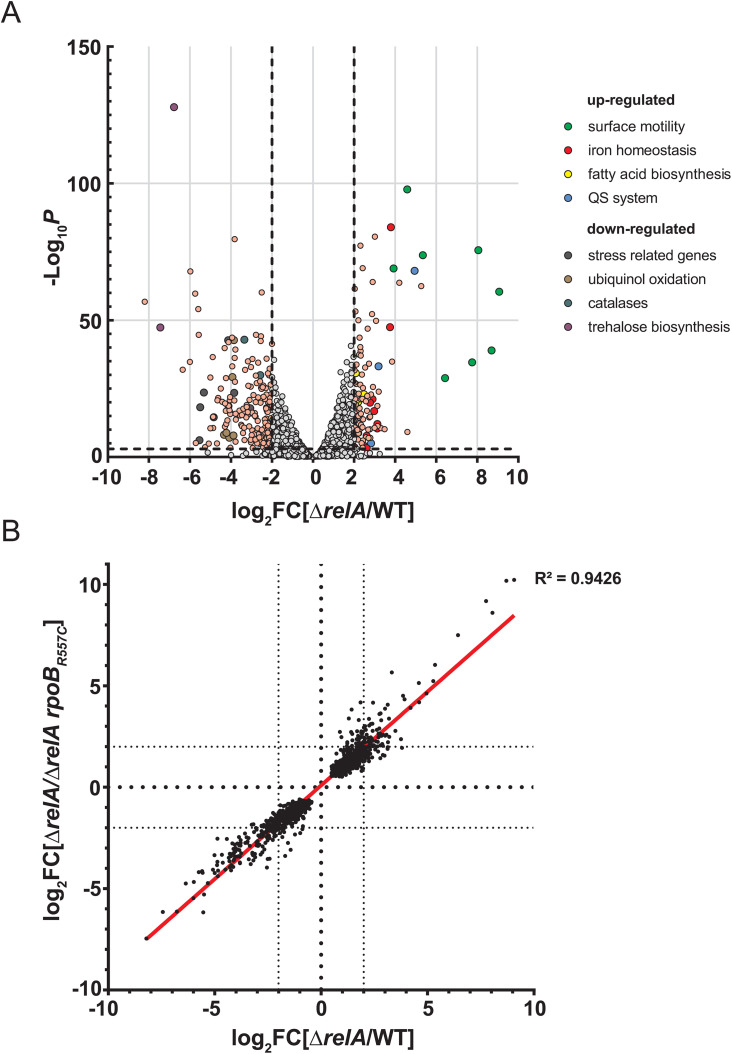
The *rpoB*_*R557C*_ mutation reverts back the transcriptomic profile of the Δ*relA* mutant close to the WT on low agar complex medium (A) Volcano plot representing the genes differentially expressed in the Δ*relA* mutant compared to the WT strain on low agar medium. Genes with a |log_2_FC| ≥ 2 and –Log_10_ *p*-value ≥ 3 are represented in light pink or with a color code for group of genes associated with known functions (light green: surface motility; red: iron homeostasis; yellow: fatty acid biosynthesis; blue: quorum sensing (QS) system; black: stress related genes; brown: ubiquinol oxidation; purple: trehalose biosynthesis; dark green: catalases). **(B)** xy-plot representing the differential expression of genes between the Δ*relA*/WT *vs* Δ*relA/*Δ*relA rpoB*_*R557C*_ on motility complex medium. Each dot represents the log_2_FC value for the strains indicated under brackets (only genes with FDR-adjusted *p* ≤ 0.005 were kept).

To further characterize the suppressing effects of *rpoB*_*R557C*_ in Δ*relA*, the RNA extracted from the WT, Δ*relA* and Δ*relA rpoB*_*R557C*_ cells grown on the medium used for the selection of the suppressors – LB low agar plates – was sequenced. When comparing Δ*relA* to the WT, we found 304 (8.09%) differentially expressed genes (DEGs) (absolute log_2_ Fold Change |log_2_FC| ≥ 2 and FDR–adjusted *p*-value ≤ 0.005), with 115 (3.06%) up-regulated genes and 189 (5.03%) down-regulated genes (S1 Table). As expected, the *ABUW_3766–3773* operon known to be critical for surface motility [24,25] was found among the most up-regulated genes in the Δ*relA* strain (Fig 7A).

Next to this operon, the *abaRMI* (*ABUW_3774–3776*) quorum sensing-related locus was also highly upregulated. Genes related to iron homeostasis (acinetobactin gene cluster and several TonB-dependent siderophore receptor) and fatty acid (FA) biosynthesis were also upregulated in the Δ*relA* mutant (Fig 7A and S1 Table). Several genes known to be regulated by (p)ppGpp in *E. coli* (*ostAB*, *raiA* and *fis*) were found to be downregulated in Δ*relA*. In addition, the expression of the locus *ABUW_2433–2443*, containing stress related genes such as *cinA1*, *katE, dtpA and dtpB* was also found to be downregulated in Δ*relA*. Apart from these, most of the other downregulated genes were of unknown functions (Fig 7A and S1 Table**).**

Interestingly, we only found 21 (0.56%) DEGs between the WT and the suppressor Δ*relA rpoB*_*R557C*_ strain, with respectively 15 (0.40%) upregulated and 6 (0.16%) downregulated genes. Fig 7B shows that the log_2_FC values of Δ*relA vs* WT and Δ*relA vs* Δ*relA rpoB*_*R557C*_ have a linear correlation (R² = 0.9426). This indicates that the transcriptomic changes induced in the Δ*relA* mutant are almost entirely reverted to WT levels in the Δ*relA rpoB*_*R557C*_ suppressor.

We also determined the (p)ppGpp-dependent transcriptome for cells grown in liquid LB medium by comparing WT to Δ*relA* or Δ*relA rpoB*_*R557C*_ strains. However, in these conditions only 153 genes (4.06%) were differentially expressed in Δ*relA* compared to WT (absolute log_2_ Fold Change |log_2_FC| ≥ 2 and FDR–adjusted *p*-value ≤ 0.005), with 28 (0.74%) upregulated genes and 125 (3.32%) downregulated genes (S6A Fig and S2 Table). All the genes encoding the assembly machinery of Csu pili (*csu* genes) were downregulated in Δ*relA* (S6A Fig). Importantly, there were still 165 DEGs when comparing the suppressor Δ*relA rpoB*_*R557C*_ to the WT strain (S2 Table), suggesting that the *rpoB*_*R557C*_ restored the (p)ppGpp-dependent transcriptional profile more efficiently on low agar plate than in liquid medium. In agreement with this, the linear regression between the log_2_FC values of Δ*relA vs* WT and Δ*relA vs* Δ*relA rpoB*_*R557C*_ did not fit well for the liquid medium (R² = 0.4531; S6B Fig), showing a low correlation. These data suggest that the contact of *A. baumannii* onto a semi-solid surface could induce (p)ppGpp accumulation, which leads to a deep transcriptional reprogramming.

### The Δ*relA* mutant is highly sensitive to desiccation and have impaired H_2_O_2_ detoxification

Among the genes differentially expressed in the Δ*relA* mutant in both liquid and low agar media, we identified the TetR-type transcriptional regulator *ABUW_1645*. It was the most downregulated gene in liquid medium (log_2_FC = -7.91) and among the most downregulated on low agar plates (log_2_FC = -5.27). This transcriptional regulator has been previously described as being involved in a pleiotropic phenotypic switch in *A. baumannii* [26]. Since its expression appears to be partially restored in the Δ*relA rpoB*_*R557C*_ suppressor strain compared to the WT (log_2_FC of -0.61 in liquid and -2.32 in low agar medium), we wondered whether it could contribute to the phenotypes observed in the Δ*relA* mutant. To test this hypothesis, a second copy of *ABUW_1645* expressed from the IPTG-inducibe P_*tac*_ promoter (P_*tac*_-*ABUW_1645*) was introduced in the Δ*relA* mutant as well as in the WT. However, this construct turned out to be lethal on IPTG containing plates, not only in the Δ*relA* mutant but also in the WT strain, while it did not impact the growth or the colony appearance without IPTG. Occasionally, WT and Δ*relA* clones with P_*tac*_-*ABUW_1645* were able to grow in the presence of IPTG, but these clones harbored IS4 ISAba1 transposase inserted into *ABUW_1645*, thereby disrupting its function. Although we cannot firmly exclude the possibility that *ABUW_1645* participates to some extent to the phenotypes displayed by Δ*relA*, our data show that *ABUW_1645* is toxic when highly expressed.

**Fig 8:**
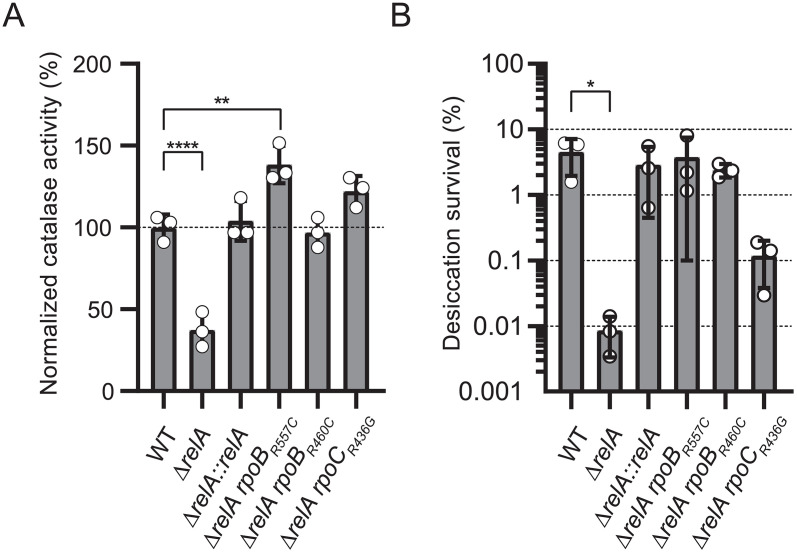
The Δ*relA* mutant has impaired catalase activity and desiccation resistance. **(A)** Catalase activity produced by overnight culture of the WT, Δ*relA,* Δ*relA*::*relA*, Δ*relA rpoB*_*R557C*_, Δ*relA rpoB*_*R460C*_ and Δ*relA rpoC*_*R436G*_ strains. ANOVA and Dunnet’s multiple comparisons test (p < 0.01 and *p* < 0.0001). **(B)** Desiccation survival of the same strains after 3 days at 35% room humidity and 25 °C. Kruskal-Wallis and Dunn’s multiple comparisons test (*p* < 0.05).

On low agar medium, the most downregulated gene in the Δ*relA* strain is *dtpA* (log_2_FC = -8.21), which encodes a hydrophilin involved in the tolerance of the *Acinetobacter calcoaceticus–baumannii* complex to high desiccation [15]. In fact, the entire *ABUW_2433–2443* locus in which *dtpA* is encoded is downregulated in both liquid and low agar media. Since this locus includes several other stress-related genes such as those encoding the second hydrophilin DtpB and the catalase KatE, we measured the tolerance to desiccation and the catalase activity in the WT, Δ*relA* and suppressors strains. The Δ*relA* strain showed a significant decrease (*p* < 0.0001) in normalized catalase activity (37.4% ± 10.6%) compared to the WT strain (100% ± 8.0%). All the three Δ*relA* suppressors displayed a normalized catalase activity close to or above the WT, with respectively 138.4% ± 11.5% (*rpoB*_*R557C*_), 97.0% ± 9.1% (*rpoB*_*R460C*_) and 122.2% ± 9.3% (*rpoC*_*R436G*_) (Fig 8A). We then tested the desiccation survival of the same strains. After three days at 35% room humidity, 4.5% ± 2.5% of CFU were recovered for the WT strain while only 0.009% ± 0.005% CFU were recovered for the Δ*relA* mutant. Compared to the Δ*relA* mutant, all the three suppressors showed increased desiccation survival although with some differences between them in the CFU recovery (3.8% ± 3.7% for *rpoB*_*R557C*_, 2.4% ± 0.6% for *rpoB*_*R460C*_ and 0.12% ± 0.08% for *rpoC*_*R436G*_) (Fig 8B).

## Discussion

In this study, we investigated and characterized the pleiotropic roles played by (p)ppGpp in *A. baumannii*. Although *A. baumannii* and *E. coli* both belong to γ-proteobacteria and share several commonalities, we found important differences in the (p)ppGpp network of both species. First, we analyzed the long RSHs in *A. baumannii*, RelA and SpoT. All attempts to generate a single *spoT* mutant failed, suggesting that SpoT is essential in *A. baumannii*, even in the presence of SahA. In all β- and γ-Proteobacteria studied so far, differences have been observed between a single Δ*relA* mutant and a double Δ*relA* Δ*spoT* mutant, essentially because SpoT is bifunctional. However, in *A. baumannii*, both RSHs are monofunctional, with RelA working as the sole (p)ppGpp synthetase and SpoT as a hydrolase [4]. We found that both Δ*relA* and Δ*relA* Δ*spoT* mutants exhibited the same phenotypes in all the tested conditions, both behaving as (p)ppGpp^0^ strains as expected. Therefore, our results exclude any (p)ppGpp-independent role played by the SpoT protein in *A. baumannii*, at least for the phenotypes described here and in our conditions. While in *E. coli*, the bifunctional (p)ppGpp synthetase/hydrolase SpoT is the only enzyme hydrolyzing (p)ppGpp, *A. baumannii* encodes a second monofunctional (p)ppGpp hydrolase. Indeed, we identified an orphan gene (*ABUW_1957*) coding for a SAH (SahA) and showed that this enzyme was able to hydrolyze (p)ppGpp both in *E. coli* and *A. baumannii*. However, under the tested conditions, its hydrolase activity alone was insufficient to counterbalance the absence of *spoT* and to counteract (p)ppGpp produced by RelA. During our phenotypic analysis of *A. baumannii* RSH enzymes, we did not identify a condition where the Δ*sahA* mutant behaved differently from the WT strain, including virulence in *G. mellonella*. Small alarmone hydrolases remain poorly characterized in bacteria but recently, two orphan SAHs have been described in Proteobacteria. In addition to (p)ppGpp, SAHs were shown to degrade another alarmone, (p)ppApp, and were suggested to act as a defensive mechanism during bacterial competition [27–29]. It is therefore tempting to speculate that SahA plays a similar role during host colonization, but the involvement of small RSHs in bacterial competition is still an emerging field, requiring further investigation. We also identified a putative orphan SAS (*ABUW_0769*) that might synthesize (p)ppGpp. However, we did not find any correlation between its expression and (p)ppGpp production neither in *E. coli* nor in *A. baumannii*. Nevertheless, we cannot rule out the possibility that a specific condition is required to activate (p)ppGpp synthesis by *ABUW_0769*.

Another important difference with *E. coli* is that the absence of (p)ppGpp in *A. baumannii* activated surface motility on low agar plates and altered colony morphology, likely reflecting a change of the cell surface properties. To expand our analysis, we also generated a (p)ppGpp^0^ strain in *A. baumannii* ATCC17978 and we obtained similar results in both AB5075 and ATCC17978 backgrounds, including the surface motility since both WT were not motile while (p)ppGpp^0^ mutants were hyper-motile. This was surprising considering that *A. baumannii* ATCC17978 WT strain was often reported in the literature as motile and a Δ*relA* mutant has been shown in one report to be less motile than the WT strain [19]. Aside from lab conditions and experimental setup, these differences could also be explained by the fact that there are at least two main laboratory variants of the *A. baumannii* ATCC17978 strain [21], the primary difference being the presence or absence of a 44-kb locus (AbaAL44) known to be responsible for several phenotypic changes including surface motility [21]. In addition to the AbaAL44 locus, several SNPs have been also identified, including a mutation in *obgE*, a GTPase that may be involved in the SR and thus have an impact on surface motility [30]. The lack of (p)ppGpp also had a detrimental effect on cellular morphology, leading to filamentation of a part of the population in stationary phase but this phenotype was already described in *E. coli*. Finally, as already observed with all pathogens studied so far, the virulence of *A. baumannii* in *G. mellonella* was also impacted in the (p)ppGpp^0^ strains.

While challenging the (p)ppGpp^0^ strains with stressful conditions known to be (p)ppGpp-dependent in other species, we found that surprisingly and in contrast to *E. coli*, *A. baumannii* (p)ppGpp^0^ cells remained prototrophic for amino acids when grown in synthetic minimal medium with xylose as sole carbon source. However, (p)ppGpp strongly accumulated upon SHX treatment, indicating that the hallmark of the SR is functional in *A. baumannii*. We also observed that FA and iron starvation both affected the growth of (p)ppGpp^0^ cells whereas only iron limitation led to (p)ppGpp accumulation. In *E. coli*, FA starvation leads first to a SpoT-dependent (p)ppGpp synthesis [31]. Given that the SpoT SD domain is degenerated and non-functional in *A. baumannii*, this could explain the lack of (p)ppGpp production upon cerulenin exposure. A prolonged FA starvation in *E. coli* ultimately leads to the depletion of pyruvate used as a precursor for lysine synthesis, which thereby triggers the (p)ppGpp synthesis by RelA [32]. However, this regulation has only been described in *E. coli* and may differ in *A. baumannii*. Notwithstanding the absence of (p)ppGpp accumulation upon FA starvation, our transcriptomic analyses show that genes coding enzymes involved in FA biosynthesis are upregulated in Δ*relA*, suggesting that (p)ppGpp^0^ cells behave like being starved for FA even in rich media. At least these data exclude the possibility that the growth defects of Δ*relA* cells in the presence of cerulenin or triclosan comes from low levels of FA biosynthetic enzymes. Iron deprivation also leads to (p)ppGpp accumulation in *E. coli* but in contrast to FA starvation, only SpoT is involved with its activity switching toward (p)ppGpp synthesis over hydrolysis [33]. In *A. baumannii* however, (p)ppGpp production during iron starvation can only result from (p)ppGpp synthesis by RelA. Two not mutually exclusive hypothetical scenarios are therefore possible. Either the SpoT-dependent regulation is conserved, which leads to a decrease of (p)ppGpp hydrolysis and a concomitant increase of (p)ppGpp levels due to basal synthetase activity of RelA, which cannot be hydrolyzed. Or iron starvation indirectly leads to amino acid(s) starvation, which in turn activates (p)ppGpp synthesis by RelA.

During the characterization of surface motility, we observed an intriguing phenomenon since after prolonged incubation, white and shiny colonies appeared on the bacterial motility lawn of (p)ppGpp^0^ cells, strikingly resembling to WT colonies. These extragenic suppressors are RNAP mutants that not only reverted surface motility but also suppressed most phenotypes associated with the loss of (p)ppGpp, including virulence in *G. mellonella*. Surprisingly, the *rpoB*_*R460C*_ mutation was as efficient as *rpoB*_*R557C*_ and *rpoC*_*R436G*_ to suppress the lack of virulence of Δ*relA* despite the very strong filamentation observed in stationary phase. Of course, we cannot rule out the possibility that this filamentation was not induced inside *G. mellonella*.

Pathogens in which (p)ppGpp production is inactivated are classically avirulent, or strongly attenuated, in different infection models. For instance, this is the case for *Staphylococcus aureus* upon zebrafish larval infection [34], *Pseudomonas aeruginosa* in *Drosophila melanogaster* [35], *Enterococcus faecalis* in *Caenorhabditis elegans* [36], or mice infected with *S. aureus* [37], *Borrelia burgdorferi* [38], *Brucella* spp. [39], *Yersinia pestis* [40] or *Salmonella enterica* [41]. Although (p)ppGpp is systematically required for virulence, the molecular reason(s) behind attenuation is still unknown and likely different from one pathogen to another. Interestingly, (p)ppGpp has also been shown in these pathogens to regulate virulence factors in laboratory conditions, such as type III secretion systems in *Y. pestis* [40] or type IV in *Brucella* spp. [39], or the pathogenicity islands in *S. enterica* [42]. However, we do not know if the lack of virulence comes from these specific defects or rather from a more general enhanced sensitivity to stressful conditions (nutrient starvation, acidic pH, …) associated with (p)ppGpp^0^ strains. Likewise, RNAP stringent mutations are able suppress growth defects of *E. coli* [11] or to restore expression of fimbriae in uropathogenic *E. coli* [43] in laboratory conditions, but these suppressors were never shown to suppress virulence defects. Here we show that RNAP stringent mutations restored virulence to a level comparable to the WT, despite the absence of (p)ppGpp. Strikingly, suppressor mutations selected in *codY* were shown to partially suppress the attenuation of *Listeria monocytogenes* (p)ppGpp^0^ strains [44]. This is particularly interesting knowing that in firmicutes, instead of binding directly on the RNAP, (p)ppGpp rather regulates transcription by inhibiting biosynthesis of GTP, which is required for CodY to be active as a transcription factor [45]. However, in contrast to *L. monocytogenes*, the inactivation of *codY* did not restore the virulence of *S. aureus* (p)ppGpp^0^ strain [34]. These data together with our work suggest that the (p)ppGpp-dependent transcriptional regulation might be shared by several pathogens to successfully invade, survive and replicate inside host.

We applied our motility suppressor screen combined with high-throughput sequencing (Mut-Seq) to isolate as many mutations as possible in RNAP. With this approach, 57 putative missense mutations were identified at 48 different positions in *rpoB* or *rpoC*, among which we found *rpoB*_*R460C*_ and *rpoB*_*R557C*_ that we had previously isolated. In 2006, Trinh and colleagues undertook the enormous task of compiling most RNAP mutations reported so far across various organisms, including *E. coli* [23]. Among these were also mutations mimicking (p)ppGpp effects, known as “RNAP stringent mutations”. The list of RNAP stringent mutations in *E. coli* was then updated by Potrykus and Cashel [46]. We compared mutations obtained with Mut-Seq to both lists and found that ~40% of the stringent mutations described in *E. coli* were also present in our conditions in *A. baumannii*, strongly suggesting that our screen was effective in selecting for stringent mutations, including 20 putative mutations in novel positions (9 in *rpoB* and 11 in *rpoC*) that we believe have never been described before.

With a total of 57 different substitutions identified within *rpoB* and *rpoC* in our screen, and in agreement with work done on *E. coli*, RNAP seems to be the primary target for mutations in the (p)ppGpp^0^ background in *A. baumannii*. In support of that, by comparing the WT, Δ*relA*, and Δ*relA rpoB*_*R557C*_ strains on surface motility medium, we found that (i) the expression of many genes (∼8%) is regulated by (p)ppGpp and (ii) their expression was mostly reverted close to the WT level with the *rpoB*_*R557C*_ mutation. In contrast, the (p)ppGpp-dependent regulon was much smaller (∼4%) when strains were sampled from mid-exponential phase of growth in liquid complex medium and *rpoB*_*R557C*_ poorly suppressed these transcriptional changes. Together, these data suggest that the low agar medium used for surface motility is “stressful” for *A. baumannii*, at least more than the liquid complex medium. In support of this, the *ABUW_2433–2444* locus containing stress-related genes linked to oxidative stress and desiccation was highly up-regulated in the WT strain (mean log_2_FC locus = 5.34 ± 2.11) grown on semi-solid medium compared to liquid medium. On the contrary, the expression of the hydrophilins (*dtpA* and *dtpB*) and the catalase (*katE*) encoded in this locus was not induced in (p)ppGpp^0^ cells, which led to a poor desiccation resistance and catalase activity. Strikingly, the suppressor mutations (*rpoB*_*R460C*_, *rpoB*_*R557C*_ and *rpoC*_*R436G*_) were all able to suppress these phenotypes, which corroborates the high expression of the *ABUW_2433–2444* locus observed in the Δ*relA rpoB*_*R557C*_ strain. The (p)ppGpp-dependent increase of catalase activity might of course help *A. baumannii* to resist to H_2_O_2_ produced in the hemolymph of *G. mellonella* [47] as well as to survive the oxidative burst generated by the innate immune system of a human patient infected with *A. baumannii*. But before potential infection, it is tempting to speculate that *A. baumannii* evolved to activate the stringent response upon contact with solid surface. Hence, the strong (p)ppGpp-dependent increase of expression of stress-related genes – including hydrophilins and catalase – could contribute to the high resilience of *A. baumannii* on hospital surfaces and equipment, as well as to resist to the oxidative stress generated by treatment with detergents. Thus, the interplay between (p)ppGpp and RNAP we describe here may be critical for the opportunistic pathogen *A. baumannii* to survive in hospital environments before potentially infecting patients.

## Materials and methods

### Bacterial strains and growth conditions

Strains, plasmids and oligonucleotides used in this study are listed in S3 Table. Details for plasmids construction are presented in S1 Methods. *E. coli* and *A. baumannii* strains were grown at 37 °C either aerobically with shaking at 175 rpm or on plate with 1.5% agar (Difco). LB Broth Base (Invitrogen) medium was used as complex medium for both *E. coli* and *A. baumannii*. M9 and MOPS were used as a synthetic minimal medium with xylose 0.3% (M9X and MOPSX, respectively) as carbon a source for *A. baumannii*. M9 was prepared using M9 salts (49 mM Na_2_HPO_4_, 22 mM KH_2_PO_4_, 8.6 mM NH_4_Cl 18.6 mM NH_4_Cl) supplemented with 1 mM MgSO_4_ and 0.1 mM CaCl_2_. MOPS was prepared as described in [48]. When needed 0.5% glucose, 0.5% arabinose or 500 μM/1mM IPTG were added.

Growth was monitored by measuring absorbance at OD_600 nm_ in liquid cultures using an automated plate reader (Biotek, Epoch 2) with continuous linear shaking (700 rpm) for at least 20 h at 37 °C. The growth curves represent an average of three technical replicates and at least three biological replicates were performed for each experiment. For the spotting assays, overnight cultures were serial diluted (1:10) in M9 salts. Then, 5 μL of cells were spotted on LB or M9 agar plates with necessary supplements and incubated at 37 °C for at least 20 h. At least two biological replicates were performed for each experiment.

For *E. coli*, antibiotics were used at the following concentrations (μg/mL; in liquid/solid medium): chloramphenicol (30/30), apramycin (30/30) while for *A. baumannii*, media were supplemented with apramycin (30/30) or rifampicin (20/20) when appropriate. Plasmid delivery into *A. baumannii* was achieved by natural transformation as described in [49]. In-frame deletions were created by using the pK18-*mobsacB* derivative plasmids as follows. Integration of the plasmids in the *A. baumannii* genome after single homologous recombination were selected on LB plates supplemented with apramycin. Independent recombinant clones were then inoculated in LB medium without antibiotics and incubated overnight (ON) at 37 °C. Then, dilutions were spread on LB agar plates without NaCl and supplemented with 10% sucrose and incubated at 30 °C. Single colonies were picked and transferred onto LB agar plates with and without apramycin. Finally, to discriminate between mutated and wild-type loci, apramycin-sensitive clones were tested by PCR on colony using locus-specific oligonucleotides.

### Microscopy

Five microliters of ON culture diluted to 1:2 were spotted on a LB agar pad. Phase contrast images were obtained using Axio Observer 7 microscope (ZEISS, Germany), Orca-Flash 4.0 camera (Hamamatsu, Japan) and Zen 3.9 software (ZEISS, Germany). Analysis was done with the MicrobeJ plugin [50] on Fiji software [51] in order to determine the cell length.

### Motility assay

Motility medium containing LB supplemented with 0.5% agar (Difco) was prepared the day prior of the experiment and plates were poured extemporaneously. A single isolated colony was used to inoculate a single plate with a sterile toothpick. Plates were incubated during 24 h at 37 °C.

### Measurement of (p)ppGpp

Bacteria were grown ON in MOPSX and diluted in MOPSX low phosphate to OD ~ 0.1 and grown until OD = 0.4-0.6 and then diluted again to OD ~ 0.1 in 500 μL of the same medium. Then radiolabeled phosphate [γ32P]-KH_2_PO_4_ was added (final concentration: 40 μCi/mL) and cells were incubated at 37 °C under agitation at 600 rpm. After 15 min, stressors (final concentrations: 3.4 mM serine hydroxamate, 400 μM 2,2’-dipyridyl, 250 μg/mL cerulenin or 2 μg/mL polymyxin B), their solvent alone (final concentration: 2% ethanol) or MOPSX low phosphate was added, and cells were incubated again for 10–30 min. To extract nucleotides, 200 μL of cells were added to 80 μL ice cold 50% formic acid and kept and ice for 20 min. Then, tubes were kept at -20 °C during at least 1 h. Nucleotides were migrated on polyethyleneimine (PEI) cellulose plate (Macherey-Nagel, Duren, Germany) in 1.5 M KH_2_PO_4_ (pH 3.4) at room temperature. TLC plates were air dried and exposed against MS Storage Phosphor Screen (GE Healthcare) overnight. Phosphor screens were finally visualized using an Amersham Typhoon (Cytiva).

### Virulence assay

The animal model used in this study is based on the larvae of the insect *Galleria mellonella.* Bacterial strains of *A. baumannii* were cultured ON in LB broth at 37 °C, harvested 5 min at 4500 rpm and washed twice in saline solution (0.9% NaCl). Cultures were adjusted to reach a density of ∼10^6^ CFU/larva and injected into the last proleg of *G. mellonella* larvae of ∼0.215 g each, using a 26 G needle (Terumo, Belgium) and a syringe pump (Thermofisher, MA, USA). The bacterial inoculum size was verified by plate counting on LB agar. Larvae were housed five per Petri dish and incubated at 37 °C for 6 days. Live and dead larvae were counted every 4 h for 48 h post-infection and then once a day for up to 6 days. Three independent experiments were performed to treat a total of 80 animals per condition. Data are presented on Kaplan-Meier curves, and log-rank statistical analysis was performed with GraphPad Prism 9.0 software.

### Suppressors and Mut-Seq

Suppressors were selected on surface motility (LB low agar) plates by incubating at least 96 h at 37 °C. Then single colonies were isolated, incubated in LB, grown ON at 37 °C. ON cultures were used to extract genomic DNA (NucleoSpin Tissue, Macherey-Nagel, Duren, Germany). For the Mut-Seq, six isolated colonies were used to inoculate 30 surface motility plates. Ten putative suppressors were picked on each plate and inoculated in 96 well plates. Each well was tested for surface motility, growth on LB agar plates with or without rifampicin. Overnight cultures of ~300 putative suppressors were done in 96 well plates, then 10 μL culture for each clone was pooled and used to extract genomic DNA. The *rpoB* and *rpoC* genes were amplified with pair of primers 4152/4153 and 4154/4155 respectively, on the genomic DNA mix, with Q5 High-Fidelity DNA polymerase (New England BioLab) according to the manufacturer’s recommendation. Both templates were mixed at a 1:1 ratio. Both Whole Genome Sequencing (suppressors) and DNA sequencing (Mut-Seq) were performed using an Illumina NextSeq 2000 (paired-end 2 x 100) instrument (Seqalis, Gosselies, Belgium). Data analysis was performed using HISAT2 and Rsamtools on Galaxy (https://usegalaxy.org) [52] and R software, respectively.

### Transcriptomic analysis

Total RNAs were extracted either from growing bacteria in liquid culture at comparable cell density (OD_600 nm_ ~ 0.5) or from bacterial colonies on motility medium. At least two independent biological replicates were made for each strain. Extraction was performed using RNeasy mini kit (QIAGEN) according to the manufacturer’s instructions. RNASeq TTRNA libraries were prepared and sequenced with Illumina NextSeq 2000 (paired-end 2x100) instrument (Seqalis, Gosselies, Belgium). NGS data were analysed using Galaxy (https://usegalaxy.org) [52] as described in [53]. Briefly, FastQC/Falco was used to evaluate the quality of the reads; HISAT2 was used to map the reads onto the AB5075 reference genome (NZ_CP008706) and generate bam files; featureCounts was used to generate counts tables using bam files and DESeq2 was used to determine differentially expressed genes. The Volcano plots were generated using GraphPad Prism 9 software.

### Catalase assay

Catalase assay was performed as described in [54]. Briefly, bacteria were grown ON, then washed and normalized to OD ~ 4. In a glass tube (15 mm diameter x 130 mm height), 100 μL of cells, 100 μL of 1% Triton X100 and 100 μL of undiluted hydrogen peroxide (27%) were mixed. The height of the foam was measured after 5 min and subtracted by the height of the control without cells.

### Desiccation assay

Bacteria were grown ON, 25 μL of cells or each strain were deposited in 3 different wells on a 24-well plate in a randomized manner. Cells were dried for 30 min in a microbiological safety cabinet and then incubated at 25 °C with 35% room humidity. Cells were finally harvested either directly after drying for the inoculum or after 3 days. To harvest cells, 300 μL of M9 salt was added in each well for 30 min to allow cell rehydration. Then cells were diluted and plated on LB agar plates to determine the number of colony forming units.

### Statistical analyses

All the statistical analyses were performed using GraphPad Prism 9 software.

## Supporting information

S1 Fig SahA can hydrolyze (p)ppGpp *in vivo* in *E. coli* while putative SAS does not seem to produce (p)ppGpp in *E. coli.* (A) Growth on LB plates supplemented with Kanamycin and Arabinose after P1 mediated transduction of a Δ*spoT*::*kan*^*R*^ lysate into *E. coli* MG1655 expressing *spoT*^*EC*^, *spoT*^*AB*^ or *sahA* from a pBAD33 vector. (B) Viability of *E. coli* MG1655 WT (hydrolase +) or Δ*relA* Δ*spoT* (hydrolase -) strains expressing *relA*^*EC*^ or *ABUW_0769* (putative SAS) from a pBAD33 vector. Overnight cultures were serial diluted (1:10), 5 μL of cells were spotted on LB plates with 0.5% of glucose or arabinose and incubated overnight at 37 °C.
(PDF)

S2 Fig RelA seems to be the sole (p)ppGpp synthetase in *A. baumannii.* Nucleotides extracted from cells treated with serine hydroxamate (SHX) for 15min were analyzed on thin layer chromatography. *A. baumannii* AB5075 WT, Δ*relA*, Δ*relA* Δ*spoT* strains are represented.
(PDF)

S3 Fig The Δ*sahA* and Δ*relA* Δ*spoT* mutants show similar phenotypes compared to the WT and Δ*relA* strains, respectively. (A) Representative growth of WT, Δ*relA*, Δ*relA* Δ*spoT* and Δ*sahA* at 37 °C in complex (LB) or minimal synthetic (M9X) medium for 20 h in 96-well plates. The growth curves represent the average of 3 technical replicates. (B) Microscopic analysis of WT, Δ*relA*, Δ*relA* Δ*spoT* and Δ*sahA* cells grown overnight at 37 °C in liquid LB complex medium. A representative picture (scale bar = 10 μm) is shown for each strain accompanied by a violin plot showing cell size distribution; the median size (μm) is indicated in red (between 2348 and 3308 cells were used for each strain). (C) Surface motility of cells described in (B) on low agar (0.5%) LB complex medium. The appearance and the color of the colonies can also be observed on the picture in the box in bottom left corners. Pictures for both experiments were taken after 24h incubation at 37 °C. (D) Viability of WT, Δ*relA*, Δ*relA* Δ*spoT*, Δ*sahA,* Δ*relA rpoB*_*R557C*_, Δ*relA rpoB*_*R460C*_ and Δ*relA rpoC*_*R436G*_ strains on minimal synthetic medium (M9X) with or without supplementation [DIP (200 μM); FeSO_4_ (50 μM); cerulenin (25 μg/mL); triclosan (0.125 μg/mL)]. Overnight cultures were serial diluted (1:10), 5 μL of cells were spotted on plates and incubated overnight at 37 °C.
(PDF)

S4 Fig Putative motility suppressors are easily selected on low agar medium in (p)ppGpp^0^ backgrounds. The WT, Δ*relA*, Δ*relA* Δ*spoT* and Δ*relA* Δ*spoT* Δ*sahA* strains were inoculated on low agar medium. After 6 days at 37 °C, white non-motile colonies growing on top of the motility lawn can be observed.
(PDF)

S5 Fig The small alarmone hydrolase *sahA* is not required for the virulence in the *Galleria mellonella* model. Kaplan-Meier survival analysis of *Galleria mellonella* larvae infected with the WT and Δ*sahA* strains. Each curve represents the average of three independent experiment for a total of 80 larvae per condition. No significant differences between the survival curve of Δ*sahA* mutant and the WT one (Log-rank (Mantel-Cox) test; *p* = 0.4022).
(PDF)

S6 Fig Lack of (p)ppGpp during exponential growth phase in complete medium has only on mild effect on the transcriptome profile. (A) Volcano plot representing the genes differentially expressed in the Δ*relA* mutant compared to the WT strain on liquid complete medium. Genes with a |log_2_FC| ≥ 2 and –Log_10_ *p*-value ≥ 3 are represented in light pink or with a color code for group of genes associated with known functions (blue: csu pili; black: stress related genes; purple: trehalose biosynthesis; dark green: catalases). (B) xy-plot representing the differential expression of genes between the Δ*relA*/WT *vs* Δ*relA/*Δ*relA rpoB*_*R557C*_ in liquid complex medium. Each dot represents the log_2_FC value for the strains indicated under brackets (only genes with FDR-adjusted *p* ≤ 0.005 were kept).
(PDF)

S1 Table RNA-seq analysis of *A. baumannii* AB5075 WT, ∆*relA* and ∆*relA rpoB*_*R557C*_ grown on LB low agar plates. baseMean: mean of normalized counts for all samples; log2FoldChange: log2 fold change (MLE); lfcSE: standard error; stat: Wald statistic; pvalue: Wald test p-value; padj: BH adjusted p-values.
(XLSX)

S2 Table RNA-seq analysis of *A. baumannii* AB5075 WT, ∆*relA* and ∆*relA rpoB*_*R557C*_ grown in LB liquid. baseMean: mean of normalized counts for all samples; log2FoldChange: log2 fold change (MLE); lfcSE: standard error; stat: Wald statistic; pvalue: Wald test p-value; padj: BH adjusted p-values.
(XLSX)

S3 Table Strains, plasmids and oligonucleotides used in this study. (XLSX)

S1 Methods Supplementary methods. (PDF)
