# Supplementary material for: Functional interplay between (p)ppGpp and RNAP in Acinetobacter baumannii
Source: PLoS Pathog. 2025 Dec 18;21(12):e1013795. doi: 10.1371/journal.ppat.1013795 (PMC12742793; doi:10.1371/journal.ppat.1013795)
Supplement: S1 Fig — (A) Growth on LB plates supplemented with Kanamycin and Arabinose after P1 mediated transduction of a ΔspoT::kanR lysate into E. coli MG1655 expressing spoTEC, spoTAB or sahA from a pBAD33 vector. (B) Viability of E. coli MG1655 WT (hydrolase +) or ΔrelA ΔspoT (hydrolase -) strains expressing relAEC or ABUW_0769 (putative SAS) from a pBAD33 vector. Overnight cultures were serial diluted (1:10), 5 μL of cells were spotted on LB plates with 0.5% of glucose or arabinose and incubated overnight at 37 °C. (PDF) [file ppat.1013795.s001.pdf]

Supplementary figures

A

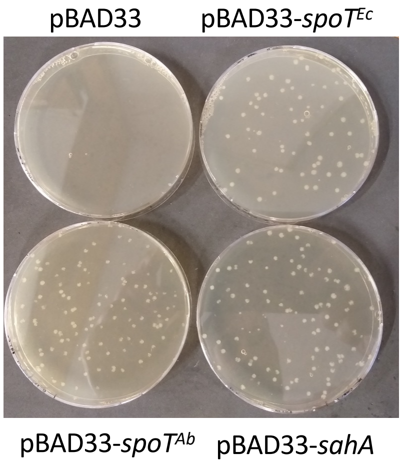

B

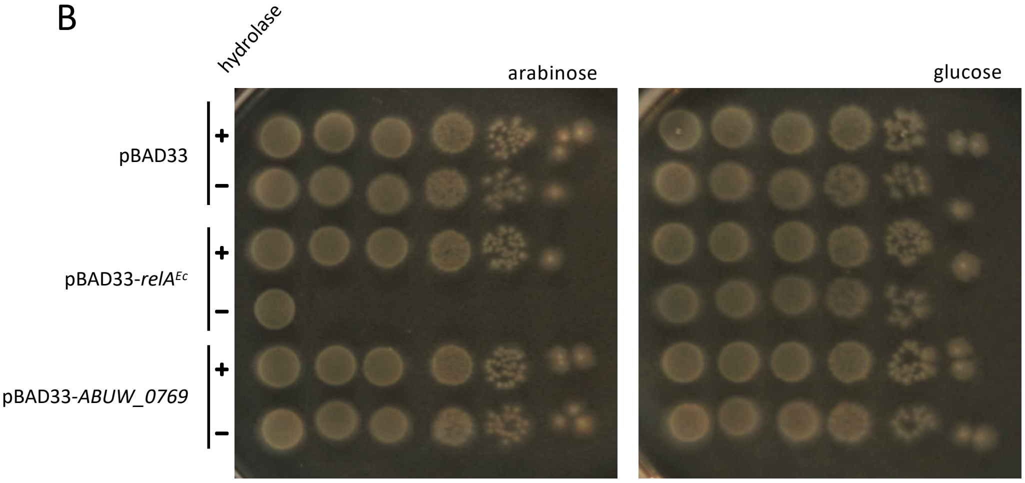

Figure S1. SahA can hydrolyze (p)ppGpp *in vivo* in *E. coli* while putative SAS does not seem to produce (p)ppGpp in *E. coli*. (**A**) Growth on LB plates supplemented with Kanamycin and Arabinose after P1 mediated transduction of a *spoT*::*kan*^{R} lysate into *E. coli* MG1655 expressing *spoT*^{Ec}, *spoT*^{Ab} or *sahA* from a pBAD33 vector. (**B**) Viability of *E. coli* MG1655 WT (hydrolase +) or Δ*relA* Δ*spoT* (hydrolase -) strains expressing *relA*^{EC} or *ABUW_0769* (putative SAS) from a pBAD33 vector. Overnight cultures were serial diluted (1:10), 5 μL of cells were spotted on LB plates with 0.5% of glucose or arabinose and incubated overnight at 37 °C.
