# Supplementary material for: Functional interplay between (p)ppGpp and RNAP in Acinetobacter baumannii
Source: PLoS Pathog. 2025 Dec 18;21(12):e1013795. doi: 10.1371/journal.ppat.1013795 (PMC12742793; doi:10.1371/journal.ppat.1013795)
Supplement: S2 Fig — Nucleotides extracted from cells treated with serine hydroxamate (SHX) for 15min were analyzed on thin layer chromatography. A. baumannii AB5075 WT, ΔrelA, ΔrelA ΔspoT strains are represented. (PDF) [file ppat.1013795.s002.pdf]

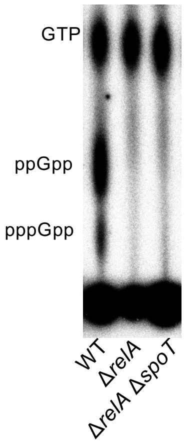

Figure S2. RelA seems to be the sole (p)ppGpp synthetase in *A. baumannii.* Nucleotides extracted from cells treated with serine hydroxamate (SHX) for 15min were analyzed on thin layer chromatography. *A. baumannii* AB5075 WT, Δ*relA*, Δ*relA* Δ*spoT strains* are represented.
