# Supplementary material for: Functional interplay between (p)ppGpp and RNAP in Acinetobacter baumannii
Source: PLoS Pathog. 2025 Dec 18;21(12):e1013795. doi: 10.1371/journal.ppat.1013795 (PMC12742793; doi:10.1371/journal.ppat.1013795)
Supplement: S3 Fig — (A) Representative growth of WT, ΔrelA, ΔrelA ΔspoT and ΔsahA at 37 °C in complex (LB) or minimal synthetic (M9X) medium for 20 h in 96-well plates. The growth curves represent the average of 3 technical replicates. (B) Microscopic analysis of WT, ΔrelA, ΔrelA ΔspoT and ΔsahA cells grown overnight at 37 °C in liquid LB complex medium. A representative picture (scale bar = 10 μm) is shown for each strain accompanied by a violin plot showing cell size distribution; the median size (μm) is indicated in red (between 2348 and 3308 cells were used for each strain). (C) Surface motility of cells described in (B) on low agar (0.5%) LB complex medium. The appearance and the color of the colonies can also be observed on the picture in the box in bottom left corners. Pictures for both experiments were taken after 24h incubation at 37 °C. (D) Viability of WT, ΔrelA, ΔrelA ΔspoT, ΔsahA, ΔrelA rpoBR557C, ΔrelA rpoBR460C and ΔrelA rpoCR436G strains on minimal synthetic medium (M9X) with or without supplementation [DIP (200 μM); FeSO4 (50 μM); cerulenin (25 μg/mL); triclosan (0.125 μg/mL)]. Overnight cultures were serial diluted (1:10), 5 μL of cells were spotted on plates and incubated overnight at 37 °C. (PDF) [file ppat.1013795.s003.pdf]

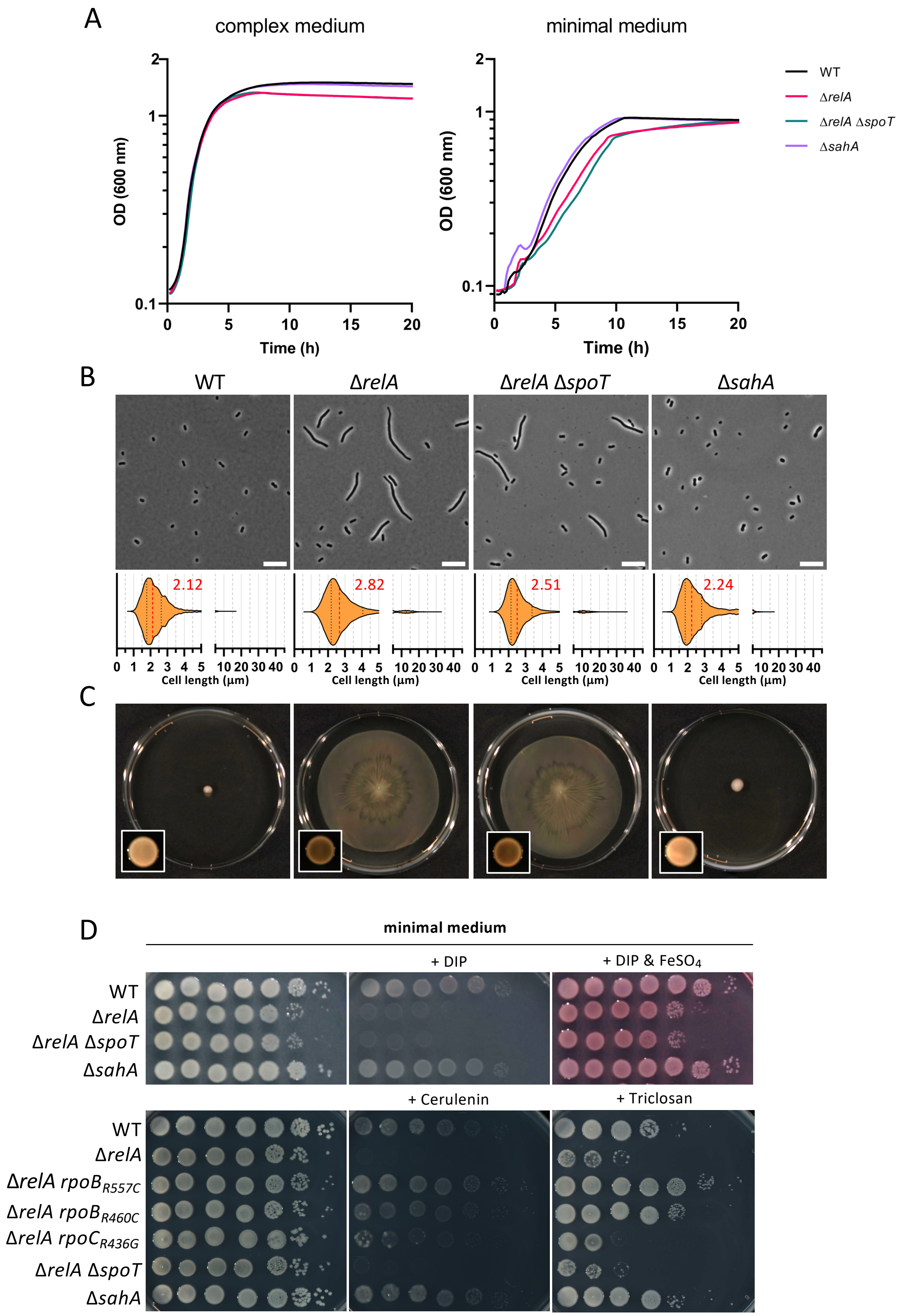

Figure S3. The Δ*sahA* and Δ*relA* Δ*spoT* mutants show similar phenotypes compared to the WT and Δ*relA* strains, respectively.(**A**) Representative growth of WT, Δ*relA*, Δ*relA* Δ*spoT* and Δ*sahA* at 37 °C in complex (LB) or minimal synthetic (M9X) medium for 20 h in 96-well plates. The growth curves represent the average of 3 technical replicates. (**B**) Microscopic analysis of WT, Δ*relA*, Δ*relA* Δ*spoT*, Δ*sahA*, Δ*relA* $rpoB_{R557C}$, Δ*relA* $rpoB_{R540C}$ and Δ*relA* $rpoC_{R436G}$ cells grown overnight at 37 °C in liquid. A representative picture (scale bar = 10 µm) is shown for each strain accompanied by a violin plot showing cell size distribution ; the median size (µm) is indicated in red (between 2348 and 3308 cells were used for each strain). (**C**) Surface motility of cells describesd in (B) on semi-solid (0.5% agar) complex medium. The appearance and the color of the colonies can also be observed in the boxed picture in bottom left corners. Pictures for both experiments were taken after 24h incubation at 37°C. (**D**) Viability of WT, Δ*relA*, Δ*relA* Δ*spoT*, Δ*sahA*, Δ*relA* $rpoB_{R557C}$, Δ*relA* $rpoB_{R540C}$ and Δ*relA* $rpoC_{R436G}$ strains on minimal synthetic medium (M9X) with or without supplementation [DIP (200 µM); $FeSO_4$ (50 µM); cerulenin (25 µg/mL); triclosan (0.125 µg/mL)]. Overnight cultures were serial diluted (1:10), 5 µL of cells were spotted on plates and incubated overnight at 37 °C.
