# Supplementary material for: Functional interplay between (p)ppGpp and RNAP in Acinetobacter baumannii
Source: PLoS Pathog. 2025 Dec 18;21(12):e1013795. doi: 10.1371/journal.ppat.1013795 (PMC12742793; doi:10.1371/journal.ppat.1013795)
Supplement: S4 Fig — The WT, ΔrelA, ΔrelA ΔspoT and ΔrelA ΔspoT ΔsahA strains were inoculated on low agar medium. After 6 days at 37 °C, white non-motile colonies growing on top of the motility lawn can be observed. (PDF) [file ppat.1013795.s004.pdf]

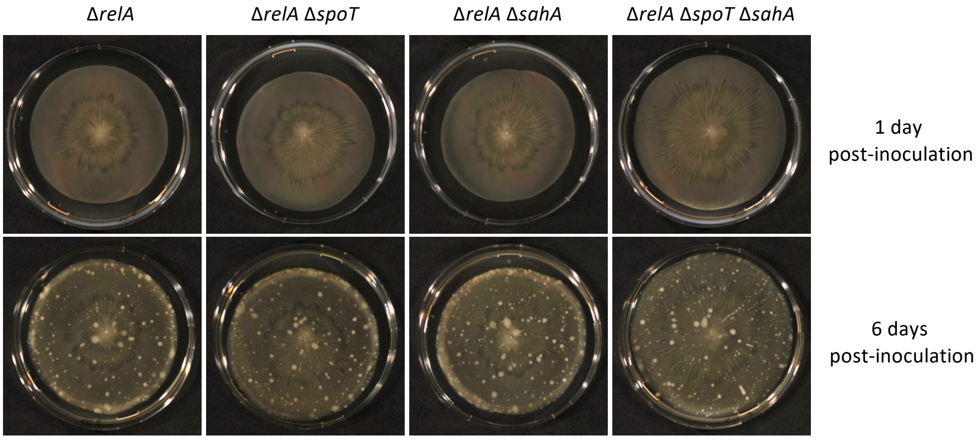

Figure S4. Putative motility suppressors easily selected on low agar medium in (p)ppGpp^0 backgrounds. The WT, Δ*relA*, Δ*relA* Δ*spoT and* Δ*relA* Δ*spoT* Δ*sahA* strains were inoculated on low agar medium. After 6 days at 37 °C, white non-motile colonies growing on top of the motility lawn can be observed.
