# Supplementary material for: Functional interplay between (p)ppGpp and RNAP in Acinetobacter baumannii
Source: PLoS Pathog. 2025 Dec 18;21(12):e1013795. doi: 10.1371/journal.ppat.1013795 (PMC12742793; doi:10.1371/journal.ppat.1013795)
Supplement: S5 Fig — Kaplan-Meier survival analysis of Galleria mellonella larvae infected with the WT and ΔsahA strains. Each curve represents the average of three independent experiment for a total of 80 larvae per condition. No significant differences between the survival curve of ΔsahA mutant and the WT one (Log-rank (Mantel-Cox) test; p = 0.4022). (PDF) [file ppat.1013795.s005.pdf]

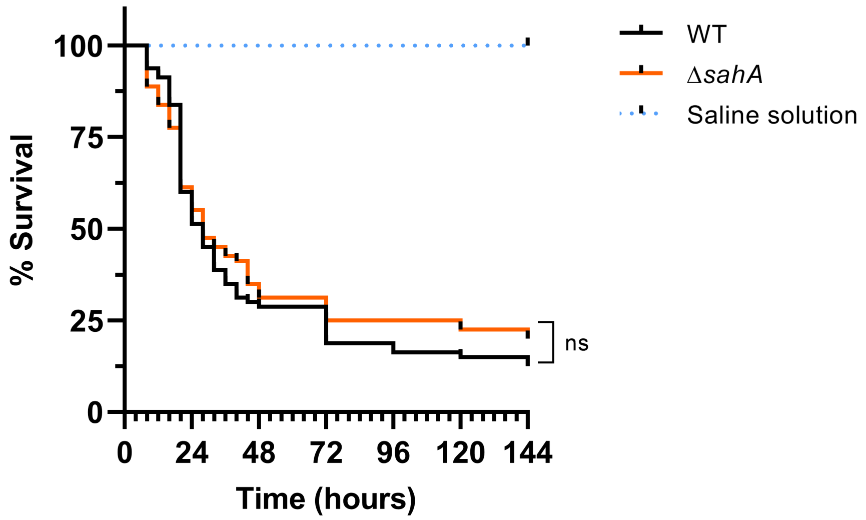

Figure S5. The small alarmone hydrolase SahA is not required for the virulence in the *Galleria mellonella* model. Kaplan-Meier survival analysis of *Galleria mellonella* larvae infected with the WT and Δ*sahA* strains. Each curve represent the average of three independent experiment for a total of 80 larvae per condition. No significant difference between the survival curve of Δ*sahA* mutant and the WT one (Log-rank (Mantel-Cox) test; p = 0.4022).
