# Supplementary material for: Functional interplay between (p)ppGpp and RNAP in Acinetobacter baumannii
Source: PLoS Pathog. 2025 Dec 18;21(12):e1013795. doi: 10.1371/journal.ppat.1013795 (PMC12742793; doi:10.1371/journal.ppat.1013795)
Supplement: S6 Fig — (A) Volcano plot representing the genes differentially expressed in the ΔrelA mutant compared to the WT strain on liquid complete medium. Genes with a |log2FC| ≥ 2 and –Log10 p-value ≥ 3 are represented in light pink or with a color code for group of genes associated with known functions (blue: csu pili; black: stress related genes; purple: trehalose biosynthesis; dark green: catalases). (B) xy-plot representing the differential expression of genes between the ΔrelA/WT vs ΔrelA/ΔrelA rpoBR557C in liquid complex medium. Each dot represents the log2FC value for the strains indicated under brackets (only genes with FDR-adjusted p ≤ 0.005 were kept). (PDF) [file ppat.1013795.s006.pdf]

A

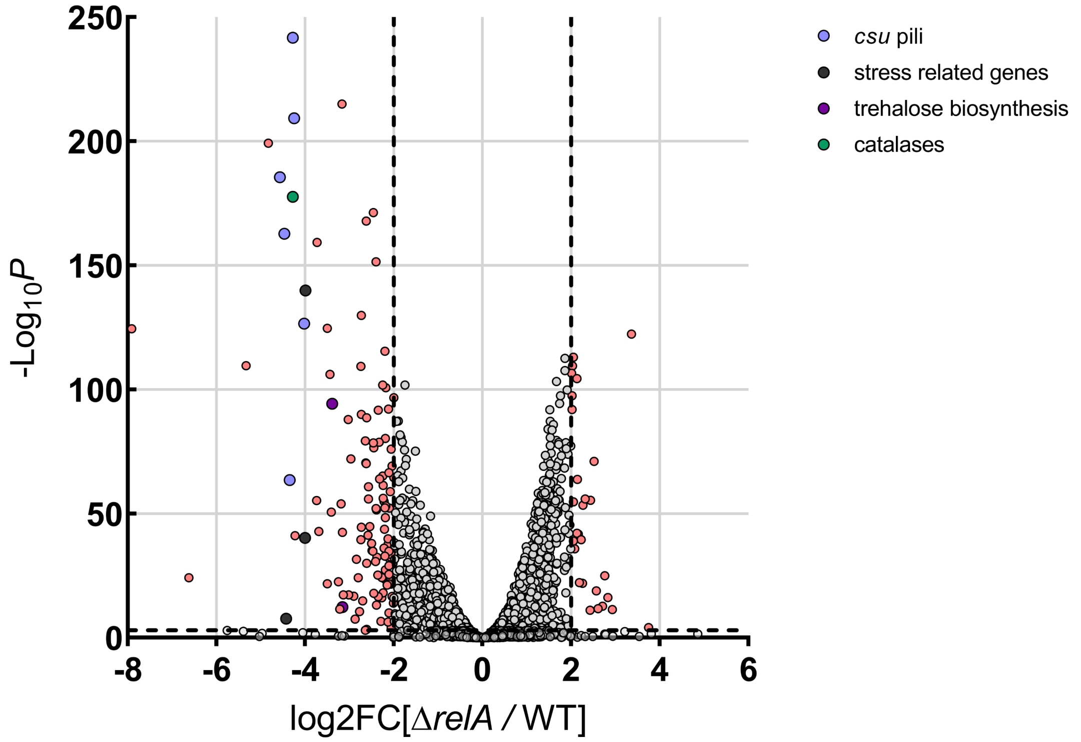

B

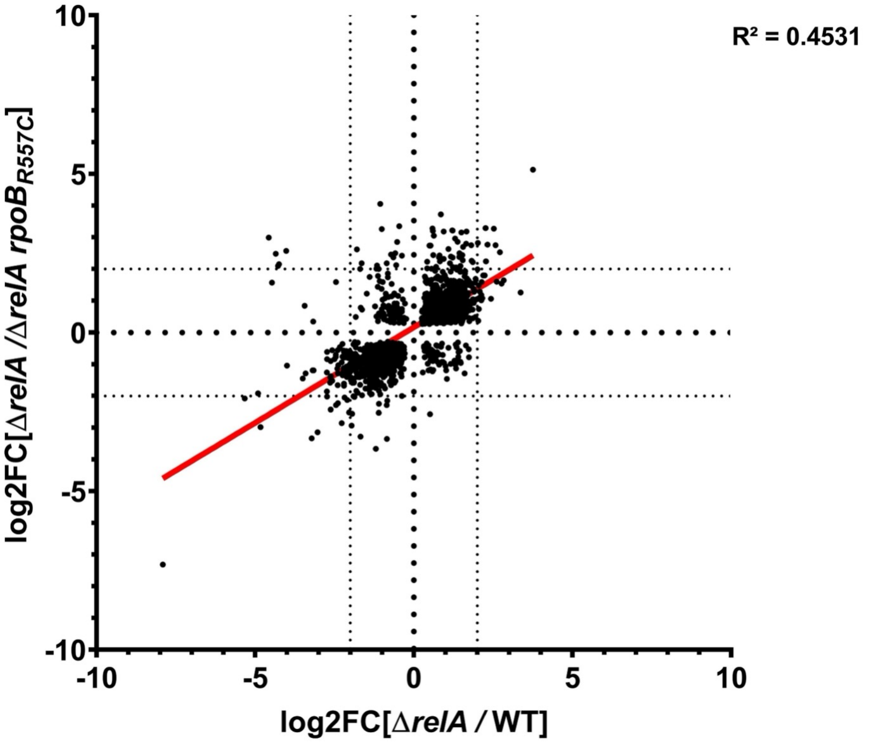

Figure S6. Lack of (p)ppGpp during exponential growth phase in complete medium has only on mild effect on the transcriptome profile. (A) Volcano plot representing the genes differentially expressed in the Δ*relA* mutant compared to the WT strain on liquid complete medium. Genes with a $|log_2FC| \geq 2$ and $-Log_{10}$ *p*-value ≥ 3 are represented in light pink or with a color code for group of genes associated with know functions (blue: csu pili; black: stress related genes; purple: trehalose biosynthesis; dark green: catalases). **(B)** xy-plot representing the differential expression of genes between the Δ*relA*/WT *vs* Δ*relA*/Δ*relA* $rpoB_{R557C}$ in liquid complex medium. Each dot represents the log_2FC value for the strains indicated under brackets (only genes with FDR-adjusted $p \leq 0.005$ were kept).
