# Supplementary material for: Functional interplay between (p)ppGpp and RNAP in Acinetobacter baumannii
Source: PLoS Pathog. 2025 Dec 18;21(12):e1013795. doi: 10.1371/journal.ppat.1013795 (PMC12742793; doi:10.1371/journal.ppat.1013795)
Supplement: S1 Methods — (PDF) [file ppat.1013795.s010.pdf]

Supplementary methods

Mut-Seq

For each nucleotide position of the *rpoB* and *rpoC* CDS, the number of sequenced nucleotides was extracted from the BAM file and expressed as percentages. Only SNPs for a given position (excluding the reference WT nucleotides) with a percentage ≥ 0.1 are represented in **Fig. 6**. Finally, we listed in **Table 1** all the putative mutations with a percentage > 0.5 and determined the corresponding codon.

Plasmids construction

To construct **pHR1189**, upstream and downstream regions of *A. baumannii* AB5075 *sahA* (*ABUW_1957*) were amplified by PCR with oligonucleotides 2780/2781 and 2782/2783, respectively. The PCR products were then digested with *Bam* HI/*Xba* I and *Xba* I/*Pst* I, respectively, and ligated into the pHR1186 vector cut with *Bam* HI and *Pst* I.

To construct **pHR1581**, upstream and downstream regions of *A. baumannii* ATCC17978 *relA* (*A1S_0579*) were amplified by PCR with oligonucleotides 2133/2134 and 2135/2136, respectively. The PCR products were then digested with *Xho* I/*Eco* RI and *Eco* RI/*Nhe* I, respectively, and ligated into the pHR1186 vector cut with *Xho* I and *Nhe* I.

To construct **pHR1190**, **pHR1191**, **pHR1192** and **pHR1584**, CDSs of *spoT*^{Ec}, *spoT*^{Ab}, *sahA* and *ABUW_0769* were amplified by PCR with oligonucleotides 2280/2281, 2282/2283, 2299/2300 and 2788/2789, respectively. The PCR products were then

digested with *Kpn* I/*Pst* I and ligated into the pBAD33 vector cut with the same restriction enzymes.

To construct **pHR1582**, the CDS of *relA*^{Ec} was amplified by PCR with oligonucleotides 4186/4187. The PCR product was then digested with *Sac* I/*Hind* III and ligated into the pBAD33 vector cut with the same restriction enzymes.

To construct **pHR1583**, the CDS of *ABUW_1645* was amplified by PCR with oligonucleotides 3518/3519. The PCR product was then digested with *Nde* I/*Bam* HI and ligated into the pHR1447 cut with the same restriction enzymes.
